# Serum prednisolone levels as a marker of oral corticosteroid adherence in severe asthma

**DOI:** 10.1186/s12890-020-01263-y

**Published:** 2020-08-27

**Authors:** J. Michael Ramsahai, Emily King, Robert Niven, Gael Tavernier, Peter A. B. Wark, Jodie L. Simpson

**Affiliations:** 1 Priority Research Centre for Healthy Lungs, Hunter Medical Research Institute, University of Newcastle, Level 2 West, Lot 1 Kookaburra Cir, New Lambton, Newcastle, NSW 2305 Australia; 2 Division of Respirology, Department of Medicine, Cumming School of Medicine, University of Calgary, Calgary, Alberta Canada; 3 North West Lung Centre, University Hospital of South Manchester, United Kingdom and Institute of Inflammation and Repair, University of Manchester, Manchester, UK

**Keywords:** Asthma, Airway markers, Eosinophils, Inflammation, Clinical respiratory medicine, Severe asthma, Biomarkers, Adherence, Prednisolone

## Abstract

**Background:**

Severe asthma is a complex heterogeneous disease typically requiring advanced therapies. Underlying the treatment of all asthma, however, is the consistent recommendation across international guidelines to ensure that adherence to therapy is adequate. Currently, there is no consensus on an objective marker of adherence.

**Methods:**

We performed a prospective observational study of 17 participants taking oral prednisolone using serum prednisolone levels as a marker of adherence, and sputum eosinophilia as a marker of control of type 2 airway inflammation. Based on these biomarkers, we classified participants into a non-adherent and an adherent cohort, and further stratified by the presence of ongoing sputum eosinophilia.

**Results:**

We identified 3 non-adherent participants and 14 who were adherent, based on their serum prednisolone levels. Stratification using sputum eosinophil counts identified one participant as having ongoing sputum eosinophilia in the setting of non-adherence, while six were identified as steroid resistant with ongoing sputum eosinophilia despite adherence to oral prednisolone therapy.

**Conclusion:**

Serum prednisolone can be used an objective marker of adherence in those patients with severe asthma taking daily oral prednisolone. In combination with sputum eosinophil counts, a steroid resistant cohort can be distinguished from one with ongoing inflammation in the setting of non-adherence. This information can then be used by clinicians to differentiate the optimal next steps for treatment in these specific populations.

**Trial registration:**

Participants were recruited as part of the Markers of Inflammation in the Management of Severe Asthma (MIMOSA) study, trial registration ACTRN12616001015437, 02 August 2016.

## Background

Asthma is estimated to affect approximately 300 million people worldwide, with an ever increasing incidence [1]. Within this staggering figure, 5–10% of this population is estimated to have severe asthma, requiring Global Initiative for Asthma (GINA) Step 4 or 5 therapy [2]. A disproportionate amount of the costs due to asthma is accrued by this particular population due to healthcare utilization, medication costs, disability, and lost productivity [3]. In addition, many of the novel biologic agents that are reserved for patients with severe asthma are relatively expensive compared to traditional therapies. As a result, it is important to distinguish truly severe asthma that is refractory to treatment from that which is difficult to control, secondary to poor inhaler technique, inadequate self-management skills, poor adherence, comorbidities, or smoking [2].

Ensuring adherence to existing therapy is essential to ensure that costly novel therapies are applied to a truly severe asthma population. The GINA guidelines [4], along with many other international guidelines have established the importance of ensuring adherence to therapy to facilitate this distinction, and ensure that any escalation of therapy is appropriate. Fraction of exhaled nitric oxide (FeNO) suppression testing has been studied to assess adherence to inhaled corticosteroids [5], however, the assessment of adherence to oral corticosteroid therapy is currently limited to self-reported or indirect outcomes. Commonly used methods include patient interview, medication diaries, pill counts, prescription fill audits, device counters, and electronic device monitors [6]. While useful, many of these options are fraught with potential sources of error. These include them being subjective, open to recall bias, as well as there being an inability to differentiate if medication is taken, absorbed, or administered appropriately. The aim of this study was to examine serum prednisolone levels as a marker of oral corticosteroid adherence in patients with severe asthma and the combination of serum prednisolone levels and sputum eosinophil counts to identify corticosteroid resistant severe asthma.

## Methods

Participants were recruited as part of the Markers of Inflammation in the Management of Severe Asthma (MIMOSA) study. (ACTRN 12616001015437) Participants were adults with severe asthma, on GINA Step 4 or 5 therapy, who continued to have uncontrolled asthma. This was defined as those patients with Asthma Control Questionnaire score (ACQ-6) > 1.5 [2, 7–9], or who required two or more courses of oral corticosteroids (OCS) for exacerbations in the past 12 months, or who required hospitalization in the past 12 months. All participants were either ex-smokers for at least 6 months, or had never smoked. In the MIMOSA study, a participant’s oral prednisolone dose was adjusted monthly based on their blood eosinophil count, fraction of exhaled nitric oxide (FeNO), or ACQ-6 score, however, no specific interventions towards adherence were applied. Participants were included in this particular sub-study if they were on a regular daily dose of prednisolone at the time of enrolment. All participants who met this criteria were included.

**Fig. 1:**
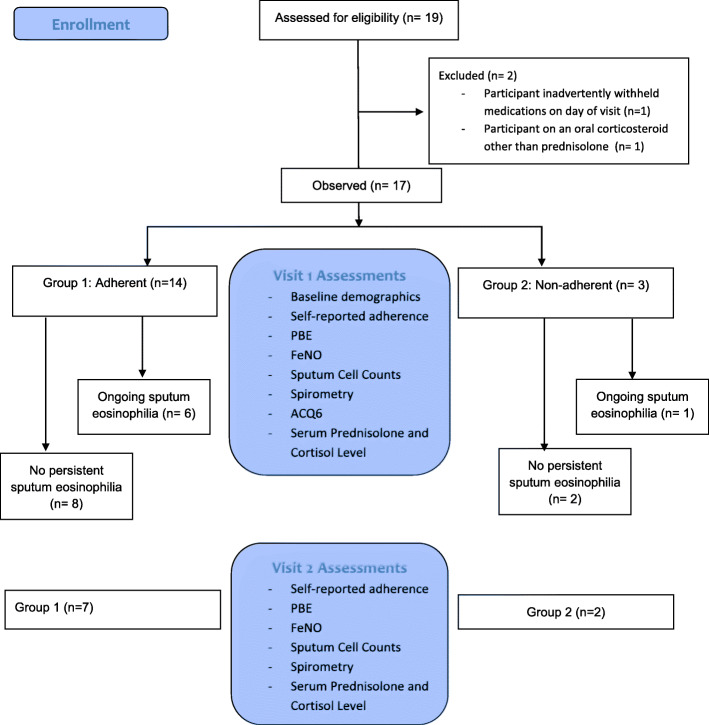
Flow chart of assessments over the course of the study

On their first visit peripheral blood and induced sputum samples were obtained, and the dose and time of last administration of their prednisolone was recorded (Fig. 1). At the end of the one-year follow-up period, these measurements were repeated. A descriptive analysis of participants who had measurements taken at both visits was performed with respect to the trends of their monthly measured values or ACQ-6 scores, blood eosinophil counts, and FeNO.

### Spirometry, FeNO, and blood eosinophil measurements

FeNO measurements were performed using a NIOX VERO (Aerocrine, Sweden) device, as per manufacturer’s suggestions and prior to spirometry. Spirometry was performed as per ATS criteria [10] using a MedGraphics CPFS/D USB-Ascensia spirometer and BreezeSuite software (Minneapolis, USA). Blood eosinophil counts were measured from samples obtained at each in-person visit through the MIMOSA study and processed at either Pathology North (NSW, Australia) laboratories, or the Hunter Medical Research Institute.

### Sputum and laboratory processing

Sputum induction and processing were performed as previously described by Gibson et al. [11] Serum samples were shipped to the University Hospital of South Manchester for analysis of serum prednisolone and cortisol levels. Serum prednisolone levels were measured using high pressure liquid chromatography tandem mass spectroscopy, as previously described [12], while cortisol measurement was performed as described by Gamble et al. [13].

A Participant was categorised as adherent if prednisolone was detectable in the serum sample. Those patients with a serum prednisolone level below the level of detection of the assay (low) were defined as non-adherent. Sputum cell count was performed as previously described [11]. Persistent eosinophilic asthma was defined as a sputum eosinophil proportion of equal to or greater than 3% [14–17]. The population of participants who were adherent with elevated sputum eosinophils, were defined as steroid resistant eosinophilic asthma [18].

Serum cortisol levels were deemed suppressed if they were less than 50 nmol/L. [19] This level has also been found to account for the effects of a short course of high dose steroids, where median cortisol is suppressed to 112 nmol/L (in a chronic obstructive pulmonary disease (COPD) population treated for 14 days) [20].

Comparisons between groups were performed using medians, interquartile ranges and Wilcoxon tests, given the small observed sample sizes. Serum cortisol, prednisolone levels over time, and proportions were compared using means, 95% confidence intervals and Student t-tests and binomial distributions. Correlations were analyzed using Pearson’s correlations.

Ethics approval was obtained from the Hunter New England Human Research Ethics (16/05/18/3.03) and University of Newcastle Human Research Ethics Committees (H-2016-0261). Written informed consent was obtained from all participants.

## Results

There were 19 participants identified on daily OCS in the MIMOSA study. One was excluded because they had inadvertently withheld their medication in preparation for the study visit, and another was excluded because they were taking oral hydrocortisone and not prednisolone (shown in Fig. 1).

The demographic information of the remaining 17 participants is summarized in Table 1. There were no significant differences in the populations between those that were adherent compared to non-adherent with respect to sex distribution, age, BMI, prednisolone dose, inhaled corticosteroid dose, exacerbation frequency, ACQ score, FEV_1_, cortisol levels, ex-smoker status, or markers of type 2 inflammation. There was higher self-reported adherence in the group defined as adherent based on serum prednisolone levels. (*p* = 0.03).

**Table 1:** Demographic features of 17 participants in whom we measured serum prednisolone levels. (Proportions are reported as absolute values and percentages, and compared using a two-sample test of proportions, while point estimates are reported as medians with interquartile ranges, and compared using Wilcoxon’s test)

Demographic	Non-adherent ***N*** = 3	Adherent ***N*** = 14	***p***-value
Sex (M)	1 (33%)	7 (50%)	0.60
Age (years)	52 [46,66]	63.5 [50,70]	0.49
Prednisolone dose (mg/day)	10 [5, 25]	6.25 [5,10]	0.35
Inhaled Corticosteroid Dose (Fluticasone Propionate equivalents in mcg)	1500 [1000,2000]	1000 [1000,1500]	0.30
Number of medications prescribed for asthma	7 [4, 7]	7 [6, 8]	0.56
BMI (kg/m^2^)	27 [16,44]	31 [27,34]	0.61
Severe Exacerbations (/year)	6 [2,10]	4 [2,6]	0.67
ACQ-6 Score	2.3 [1.3,2.8]	2.4 [1.5,3.8]	0.75
FEV_1_ (% predicted)	104 [83,105]	69 [41,83]	0.06
FeNO (ppb)	30 [13,225]	19 [12,41]	0.45
Sputum eosinophils, %	1 [0.25,9.86]	1.25 [0,10.25]	0.80
Blood Eosinophils (cellsx10^9^/L)	0.2 [0,0.5]	0.1 [0,0.116]	0.37
Cortisol level (μmol/L) (mean and 95% confidence interval)	155.2 (− 187,498)	33.0 (14.3, 51.7)	0.005
Self-reported Adherence	100% [71.4, 100]	100% [100,100]	0.03
Ex-smokers	1 (33%)	4 (29%)	0.87

Of these 17 participants, three (17.6%) were defined as non-adherent, (Table 2) one of which self-reported adherence of 71% in the last month, while the others self-reported 100% adherence. The remaining 14 (82.4%) participants were categorised as adherent and all self-reported adherence of 100% in the last month. Of the 14 adherent participants, six (42.9%) were categorised as having eosinophilic asthma with ongoing sputum eosinophilia and active type 2 inflammation, despite treatment. Markers of type 2 inflammation were significantly different when compared with those who did not have ongoing sputum eosinophilia (blood eosinophil count (cellsx10^9^/L) of 0.20 vs. 0.02, *p* = 0.003, and elevated FeNO (in parts per billion (ppb)) of 43 vs. 12, *p* = 0.002) (Table 3).

**Table 2:** Participants stratified by serum prednisolone level and sputum eosinophil count. Labels denote ongoing or persistent eosinophilia on current therapy

	Non-adherent	Adherent	Total
**Non-eosinophilic**	2	8	10
**Eosinophilic**	1	6 **Steroid Resistant**	7
**Total**	3	14	17

**Table 3:** Comparison of inflammatory markers (peripheral blood eosinophil count (PBE), and FeNO) between adherent participants with and without ongoing sputum eosinophilia. Sample medians and interquartile ranges are presented and compared using Wilcoxon’s test

	Adherent participants without ongoing sputum eosinophilia (***n*** = 8)	Adherent participants with ongoing sputum eosinophilia (steroid resistant) (***n*** = 6)	***p***-value
**PBE (cellsx10**^**9**^**/L)**	0.02 [0, 0.08]	0.20 [0.2, 0.4]	0.003
**FeNO (ppb)**	12 [9.5, 15.5]	43 [41, 48]	0.002

**Fig. 2:**
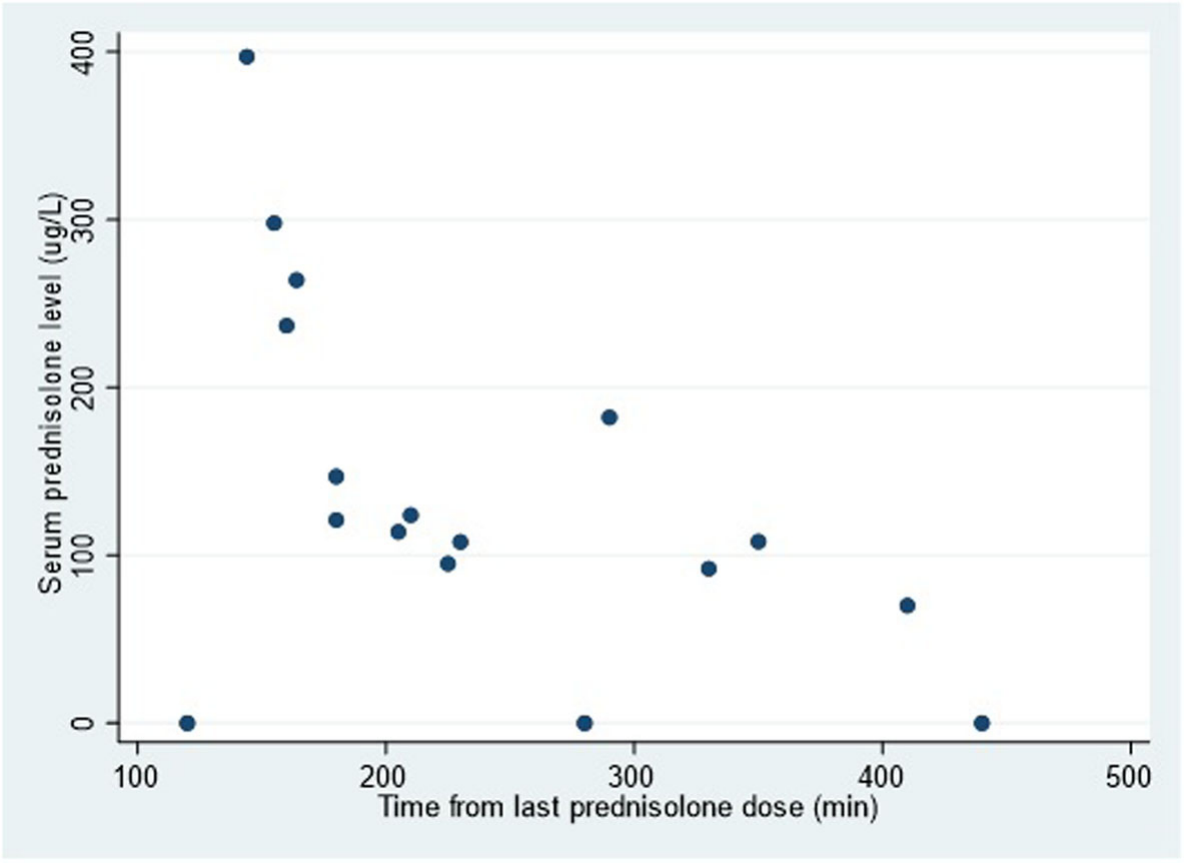
Serum prednisolone levels over time from last prednisolone dose demonstrating the expected exponential decay of serum prednisolone levels due to metabolism. (Pearson’s correlation, r = − 0.71, *p* = 0.0038)

Serum prednisolone levels were compared to the time interval between self-reported last prednisolone dose and time of blood sample collection for serum prednisolone measurement, and there was a significant association based on the expected pharmacokinetics of exponential decay (Pearson’s correlation r = − 0.72, *p* = 0.004, Fig. 2).

Mean serum cortisol levels were significantly lower in oral corticosteroid adherent compared with non-adherent participants (33.0 nmol/L versus 155.2 nmol/L, *p* = 0.005). This difference persisted at the final visit after 1 year of follow-up for the comparison of the adherent group (15.3 nmol/L), versus non-adherent (262.5 nmol/L, *p* = 0.016).

**Fig. 3:**
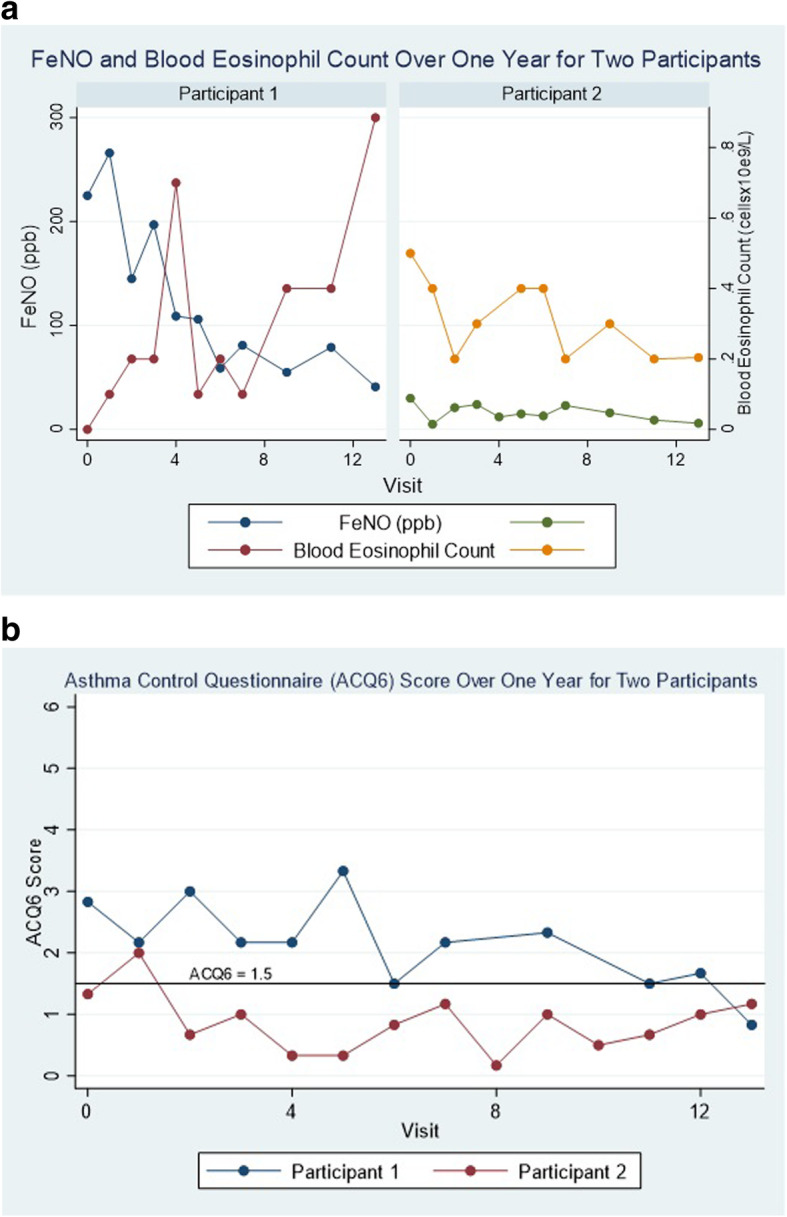
Markers of Inflammation (FeNO and Blood Eosinophil Count) (3A) and ACQ-6 (3B) over one year in a participant defined as non-adherent at Visit 0 and 13 (Participant 1) compared to one that was non-adherent initially (at Visit 0) but became adherent by Visit 13 (Participant 2)

Of the three participants who were non-adherent at the beginning of the study, one was lost to follow-up. One participant remained non-adherent at the end of the study (Participant 1), while the other became adherent at some point during the treatment regimen (Participant 2). FeNO and blood eosinophil count improved through the study for Participant 2, and ACQ-6 remained below 1.5 for the majority of the time. While FeNO also trended down for Participant 1, conversely, ACQ-6 remained at or above 1.5 throughout the study, while blood eosinophil count trended up (Fig. 3).

## Discussion

Non-adherence to OCS in this cohort with severe asthma was uncommon (3/17, 17.6%). This was lower than expected, with prior reports of up to 45% of patients on oral steroids being non-adherent [13]. We suspect that adherence may be higher in this truly severe asthma cohort given that patients would be more inclined to adhere to therapy if they are experiencing more symptoms and exacerbations. In addition, our cohort was recruited from a specialist centre for severe asthma, where participants are followed long term, and may have already benefitted from proven interventions to improve adherence. One participant was observed to have ongoing sputum eosinophilia in the setting of non-adherence where further patient education may be warranted. This participant also self-identified as being non-adherent. Of the adherent participants, six (6/14, 42.9%) had ongoing sputum eosinophilia and may be considered to have steroid refractory eosinophilic asthma. These participants could also be distinguished by difference in blood eosinophil counts, and FeNO, making this more widely applicable given real-world test availability.

The differentiation of these two populations is important since uncontrolled asthma in an adherent patient in the setting of ongoing type 2 inflammation represents a decision point for treatment escalation. This could include escalation of the dose of OCS, the addition of a long-acting anticholinergic agent (LAMA), a macrolide, or a monoclonal antibody, as per GINA guidelines [4]. In our population these participants were on either 5 mg or 7.5 mg of daily prednisone, but, half were not currently on a LAMA, and only one was currently on a biologic.

In an era of increasing expenditures related to novel agents, and, in particular, where a precision medicine approach is desired, ensuring that novel therapies are started on the right patients at the right time is crucial. This will avoid the use of futile therapies, along with their costs and side effects, in a population where alternatives may exist. Agusti et al. have proposed the concept of treatable traits in order to identify objective targets for treatment in asthma using particular biomarkers [21]. This treatable trait approach seeks to operationalize precision medicine. Using serum prednisolone as a marker of adherence would allow us to objectively distinguish our non-adherent participant from the steroid-resistant population, particularly where treatment decisions in the biologic era are concerned. While the development of novel biologic therapies has reduced the role of OCS in this population, it has not eliminated their use altogether [22–25]. As such, many patients remain on oral prednisolone and there is, thus, utility in an objective measure of adherence in this difficult to control population.

In the case of non-adherence, then, targeted interventions directed towards improving adherence could be considered before an escalation of therapy. This may include further disease-specific or medication education or counselling, reinforcement, reminders, regular assessment of asthma control and satisfaction, community managed or supervised care, the involvement of case workers, or addressing other reasons for non-adherence. Other reasons that could contribute to non-adherence pertain to the healthcare provider, health system structural, and patient factors including social inequity and financial constraints, impaired drug absorption, or treatment regimen intolerability [6, 26].

In terms of our choice of an undetectable level of serum prednisolone being used as a marker of non-adherence, this minimizes the false positive rate of our biomarker. In clinical practice, this would be important in order to minimize the misclassification of patients where an attempt may be made to pursue adherence first before any escalation in therapy. In the studied population, as the interquartile range of prednisolone dosages ranged from only 5 to 10 mg/day, serum levels across the population were largely dependent on the latency between the time of last dose and that of phlebotomy. As a result the lowest levels recorded also have the longest latency. As can be seen, in this population, the observed serum prednisolone levels plotted against time from last dose approximates the expected exponential curve of pharmacokinetic decay of serum prednisolone over time (Fig. 2) [27]. There were no medication interactions that were relevant. The use of serum prednisolone levels as a biomarker is further supported biologically by the expected development of cortisol suppression in those patients with measurable levels of prednisolone. This is an expected physiologic effect in those patients on systemic corticosteroids, and has been used in other studies where serum prednisolone is used as a marker of adherence [28, 29].

Furthermore, in the group of participants that did not have ongoing sputum eosinophilia, in spite of their poorly controlled asthma, eight were adherent and two were non-adherent, based on our definition. The use of these two measures again allows for the distinction of two different treatment avenues dependent on these markers. In the non-adherent cohort, these patients may be unnecessarily prescribed OCS, and so the opportunity exists to withdraw them, to see if type 2 inflammation returns, and consider other treatment options to improve asthma control. Given the side-effect burden of systemic corticosteroids [30], this could represent a significant benefit for this group. In the eight adherent participants without evidence of ongoing eosinophilia, treatment decisions geared toward their persistent lack of asthma control must be further contemplated based on other factors – such as treating non-type 2 inflammation or comorbidities. An assessment of airway inflammation prior to the initiation of any corticosteroid treatment was not available, so it is possible that some of these participants have only non-type 2 inflammation.

While only two non-adherent participants had measurements completed at both the initial and final visits, the pattern of their ACQ-6 scores and markers of inflammation is interesting. Participant 1 remained non-adherent to oral prednisolone through the study and this correlated with a persistently elevated ACQ-6, and blood eosinophilia. The downward trend in FeNO for Participant 1 could reflect improved adherence to inhaled therapy as part of an observer effect due to participation in the study, contrasting with adherence to oral therapy. Meanwhile, Participant 2 demonstrates improvement in FeNO, blood eosinophil counts, and a persistently well-controlled ACQ-6, giving credence to the change in their adherence status from non-adherent to adherent over the course of the study. Longitudinal assessment of these measures over time in a larger cohort would be helpful to delineate this in the future.

The low sample sizes recorded in each group make wider extrapolation difficult, but certainly justify additional study in a larger population. Given the relatively short half-life of prednisolone, serum prednisolone measures provide only a 1-day snapshot of adherence. This produces a double-edged sword where a low measure of serum prednisolone may not actually denote long-term non-adherence. Indeed, in one participant who would be deemed non-adherent due to a low serum prednisolone level, as per our definition, clinically the participant appeared adherent: serum cortisol levels were suppressed, and the participant did appear cushingoid. Other factors that may also lead to a low serum prednisolone level despite adherence on an individual basis include impaired absorption or altered metabolism. In contrast, it is difficult to distinguish whether a measureable serum prednisolone level merely reflects adherence on the day of study. This is particularly important where a participant would otherwise fall under the steroid resistant label. In our study our participants were not aware their adherence was being assessed on their first visit, but this could pose a problem in the real world. Repeated measures taken in conjunction with other methods of assessment for adherence would be important to be able to distinguish this. In addition, this study did rely on participants to accurately self-report the administration and timing of their dose of prednisolone. Finally, measurement of serum prednisolone levels is not widely available in clinical practice. Further study exemplifying the utility of this measurement as an objective marker of adherence, however, could allow for more widespread availability of testing.

## Conclusion

In conclusion, our study demonstrates that serum prednisolone levels could be useful as a measure of adherence to oral prednisolone therapy. In conjunction with sputum eosinophil counts, this can be used to help distinguish non-adherent from steroid resistant T2-high populations, in addition to a population where the opportunity may exist for steroid reduction. This provides an objective method for the assessment of adherence and can help guide clinicians on the next steps in treatment for this challenging population. With further study, serum prednisolone levels could be used to target adherence as a treatable trait and further the concept of precision medicine in severe asthma.

## Data Availability

The datasets used and/or analysed during the current study are available from the corresponding author on reasonable request.

## Abbreviations

GINA: Global Initiative for Asthma
FeNO: Fraction of exhaled nitric oxide
MIMOSA study: Markers of Inflammation in the Management of Severe Asthma study
OCS: Oral corticosteroids
ACQ-6: Asthma Control Questionnaire score
COPD: Chronic obstructive pulmonary disease
LAMA: Long-acting anticholinergic agent
ppb: Parts per billion
